# Supplementary material for: EnzML: multi-label prediction of enzyme classes using InterPro signatures
Source: BMC Bioinformatics. 2012 Apr 25;13:61. doi: 10.1186/1471-2105-13-61 (PMC3483700; doi:10.1186/1471-2105-13-61)
Supplement: Addtional file 5 — The Java code to format the data files, evaluate and predict. The file enzml_java_code.tar.gz contains the Java code used to format database data to ARFF and XML formats, to execute cross and train-test (jackknife) evaluations and to record evaluation results to database. More information is included in the readme.txt file and the Javadoc files. The code can be used with a MySQL database. To use a different database software, other JDBC drivers might be required. [file 1471-2105-13-61-S5.gz › java_code/enzml2011/doc/uk/ac/ed/inf/csb/taweka/ArffPropsTableManager.html]

ArffPropsTableManager


---


|  |  |  |
| --- | --- | --- |
| **Overview** \| **Package** \| **Class** \| **Use** \| **Tree** \| **Deprecated** \| **Index** \| **Help** | |  |
| **PREV CLASS**   **NEXT CLASS** | **FRAMES**    **NO FRAMES**     **All Classes** |  |
| SUMMARY: NESTED \| FIELD \| CONSTR \| METHOD | DETAIL: FIELD \| CONSTR \| METHOD |  |


---


## uk.ac.ed.inf.csb.taweka Class ArffPropsTableManager

```
java.lang.Object
  uk.ac.ed.inf.csb.utils.database.DbManaged
      uk.ac.ed.inf.csb.utils.database.TableManager
          uk.ac.ed.inf.csb.taweka.ArffPropsTableManager
```

---

``` public class ArffPropsTableManager extends uk.ac.ed.inf.csb.utils.database.TableManager ```

Writes into a database table the pointers needed to build an arff file (the
name of the arff file, the query to select instances, attributes, class
values etc)
TODO integrate arff recording to database with mulan experiment recording

**Version:**
:   16 Jun 2010

**Author:**
:   Luna De Ferrari luna.deferrari-at-ed.ac.uk

---

**Field Summary**

**Fields inherited from class uk.ac.ed.inf.csb.utils.database.TableManager**

`m_writer`


**Constructor Summary**

`ArffPropsTableManager(ArffGenerator arffGenerator)`
  
          Constructor 1: initialises the table (uses given database connection
file)


| **Method Summary** | |
| --- | --- |
| `uk.ac.ed.inf.csb.utils.database.TableRow` | `createRow()` |
| `ArffGenerator` | `getArffGenerator()` |
| `java.lang.String` | `getArffPathFrobDb(int recordId)` |
| `int` | `getArffRecordId()` |
| `java.lang.String` | `getPropertyValueFromTable(java.lang.String propertyName, int id)` |
| `static void` | `main(java.lang.String[] args)`             Main for recreating table |
| `void` | `writeArffRecordToDb()` |

**Methods inherited from class uk.ac.ed.inf.csb.utils.database.TableManager**

`getTableCreator, getTableName, getTableReader, getTableWriter`

**Methods inherited from class uk.ac.ed.inf.csb.utils.database.DbManaged**

`getDbConnection, getDbManager`

**Methods inherited from class java.lang.Object**

`equals, getClass, hashCode, notify, notifyAll, toString, wait, wait, wait`

**Constructor Detail**

### ArffPropsTableManager

```
public ArffPropsTableManager(ArffGenerator arffGenerator)
```

:   Constructor 1: initialises the table (uses given database connection
    file)

    **Throws:**: `java.sql.SQLException`


**Method Detail**

### createRow

```
public uk.ac.ed.inf.csb.utils.database.TableRow createRow()
```

---


### getArffGenerator

```
public ArffGenerator getArffGenerator()
```

---


### getArffPathFrobDb

```
public java.lang.String getArffPathFrobDb(int recordId)
```

---


### getArffRecordId

```
public int getArffRecordId()
```

---


### getPropertyValueFromTable

```
public java.lang.String getPropertyValueFromTable(java.lang.String propertyName,
                                                  int id)
```

---


### writeArffRecordToDb

```
public void writeArffRecordToDb()
```

---


### main

```
public static void main(java.lang.String[] args)
```

:   Main for recreating table

    :   **Parameters:**: `args` - **Throws:**: `java.sql.SQLException`


---


|  |  |  |
| --- | --- | --- |
| **Overview** \| **Package** \| **Class** \| **Use** \| **Tree** \| **Deprecated** \| **Index** \| **Help** | |  |
| **PREV CLASS**   **NEXT CLASS** | **FRAMES**    **NO FRAMES**     **All Classes** |  |
| SUMMARY: NESTED \| FIELD \| CONSTR \| METHOD | DETAIL: FIELD \| CONSTR \| METHOD |  |


---
